# Comparative Physiological and Transcriptomic Mechanisms of Defoliation in Cotton in Response to Thidiazuron versus Ethephon

**DOI:** 10.3390/ijms24087590

**Published:** 2023-04-20

**Authors:** Baopeng Liao, Fangjun Li, Fei Yi, Mingwei Du, Xiaoli Tian, Zhaohu Li

**Affiliations:** 1 Engineering Research Center of Plant Growth Regulator, Ministry of Education & College of Agronomy and Biotechnology, China Agricultural University, Beijing 100193, China; liaobaop@163.com (B.L.); lifangjun@cau.edu.cn (F.L.); yifei56@cau.edu.cn (F.Y.); dumingwei@cau.edu.cn (M.D.); lizhaohu@cau.edu.cn (Z.L.); 2 State Key Laboratory of Plant Physiology and Biochemistry, China Agricultural University, Beijing 100193, China

**Keywords:** leaf abscission, cytokinin, brassinosteroid, auxin transport, transcription factors, cotton

## Abstract

Thidiazuron (TDZ) is a widely used chemical defoliant in cotton and can stimulate the production of ethylene in leaves, which is believed to be the key factor in inducing leaf abscission. Ethephon (Eth) can also stimulate ethylene production in leaves, but it is less effective in promoting leaf shedding. In this study, the enzyme-linked immunosorbent assays (ELISA) and RNA-seq were used to determine specific changes at hormonal levels as well as transcriptomic mechanisms induced by TDZ compared with Eth. The TDZ significantly reduced the levels of auxin and cytokinin in cotton leaves, but no considerable changes were observed for Eth. In addition, TDZ specifically increased the levels of brassinosteroids and jasmonic acid in the leaves. A total of 13 764 differentially expressed genes that specifically responded to TDZ were identified by RNA-seq. The analysis of KEGG functional categories suggested that the synthesis, metabolism, and signal transduction of auxin, cytokinin, and brassinosteroid were all involved in the TDZ-induced abscission of cotton leaves. Eight auxin transport genes (*GhPIN1-c_D*, *GhPIN3_D*, *GhPIN8_A*, *GhABCB19-b_A*, *GhABCB19-b_D*, *GhABCB2-b_D*, *GhLAX6_A*, and *GhLAX7_D*) specifically responded to TDZ. The *pro35S::GhPIN3a::YFP* transgenic plants showed lower defoliation than the wild type treated with TDZ, and YFP fluorescence in leaves was almost extinguished after treatment with TDZ rather than Eth. This provides direct evidence that GhPIN3a is involved in the leaf abscission induced by TDZ. We found that 959 transcription factors (TFs) specifically responded to TDZ, and a co-expression network analysis (WGCNA) showed five hub TFs (*GhNAC72*, *GhWRKY51*, *GhWRKY70*, *GhWRKY50*, and *GhHSF24*) during chemical defoliation with TDZ. Our work sheds light on the molecular basis of TDZ-induced leaf abscission in cotton.

## 1. Introduction

Organ shedding is a common phenomenon in plants. Plants can detach diseased or senescent organs by degrading cell walls through specific mechanisms that occur in a region called the abscission zone (AZ) [1]. The AZ typically contains 5–50 layers of specialized cells, the exact number of which depends on the plant species. These cells are located at the boundary between the organ to be shed and the parent plant [2,3]. The formation of AZ is a prerequisite for plant organ abscission; however, it does not mean that the organs will drop immediately after AZ has been formed [4]. Specific signals are required from the distal part to activate the process of plant organ abscission, but the exact mechanism is not yet well understood.

The plant hormone auxin plays a significant role in the timing of abscission [5]. Studies have shown that removing the auxin source tissue promotes abscission, whereas applying auxin to the distal part blocks abscission [6,7,8,9]. It was also reported that floral organ abscission caused by aging usually occurs after a decrease in endogenous IAA levels [10]. The role of auxin in regulating abscission has been further enhanced by advances in genetic, molecular, and biochemical methods [11]. The abscission of Arabidopsis flowers has been shown to require a decrease in the auxin levels of the AZ cells, which was demonstrated by expressing either IAA-Lys synthetase or IAA biosynthetic gene under the AZ-specific polygalacturonase (PG) promoter [12]. In tomato, *SlPIN1* silencing enhanced pedicel abscission by promoting auxin accumulation in the ovary and lowering auxin levels in the AZ, indicating that auxin transport controls auxin balance to impact pedicel abscission [13]. Peng et al. [14] found that 2,4-D spraying on the litchi canopy reduced the mRNA level of *LcPG1*, which in turn resulted in a reduced fruit shedding rate.

Ethylene, as a highly mobile gaseous plant hormone, was considered an important component in activating the separation of the AZ cells [15]. In cotton, leaf explants fumigated with ethylene increased the production of endogenous ethylene synthase and the expression of abscission-specific cellulose gene *GhCel1* in the leaf abscission zone [16]. Ethephon, an ethylene-releasing chemical, has been reported to efficiently accelerate fruit abscission in olives [17], mango [18], and apple [19,20]. However, ethylene is not the only regulatory factor in abscission. Arabidopsis mutants deficient in ethylene sensing or signal transduction pathway exhibited delayed abscission of floral organs but not complete inhibition [21,22]. MacDonald et al. [23] found that balsam fir abscission still occurred even when ethylene biosynthesis or signaling was prevented by aminoethoxyvinylglycine (AVG) or 1-methylcyclopropene (1-MCP). Najeeb et al. [24] observed that cotton released less ethylene under high temperature stress but still suffered severe boll abscission. These reports led to the conclusion that factors other than ethylene may be involved in controlling organ abscission.

Cotton (*Gossypium hirsutum*) is a major commercial crop grown for its high-quality natural fibers. Machine-picking currently dominates cotton production because of changes in agricultural practices and the promotion of agricultural modernization [25]. The presence of cotton leaves often greatly reduces the efficiency of harvesting and increases the impurity content of mechanical harvesting. Therefore, an efficient chemical defoliation technology is a key measure for ensuring the quality of mechanically harvested cotton.

Thidiazuron (TDZ, a synthetic cytokinin-like chemical) is a defoliant frequently used to aid mechanical cotton harvesting. Previous physiological studies have demonstrated that TDZ promoted leaf abscission in cotton by stimulating ethylene production in the leaves and inhibiting the transport of auxin from leaves to the abscission zone [26,27]. Recently, Xu et al. [28] used RNA-seq technology to compare the differences in the response of various cotton varieties to the mixed application of TDZ and ethephon (boll opener) and found that the crosstalk between cytokinin and ethylene played a role in the regulation of defoliation. Li et al. [29] also conducted transcriptome analysis of cotton leaves, petioles, and AZ tissues after TDZ treatment, and reported that several hormones, including ethylene, auxin, and cytokinin, were involved in the TDZ-induced leaf abscission. Shu et al. [30] reported that the responses of ethylene and auxin synthesis and their signaling pathways to TDZ in the AZ were inhibited by low temperatures. However, the exact molecular mechanism of the defoliation induced by TDZ remains poorly understood.

It is interesting that both the defoliant, TDZ, and the boll opener, ethephon, could induce high levels of ethylene production in cotton leaves [26,31]. However, the latter does not lead to efficient leaf shedding. Therefore, we hypothesized that TDZ might have a specific gene regulatory profile for inducing leaf abscission, in addition to stimulating ethylene production in leaves, such as ethephon.

In this study, RNA-seq was used to investigate the transcriptome differences between TDZ-induced leaf abscission and ethephon-treated cotton leaves. Differentially expressed genes that specifically responded to TDZ (TDZ-SRGs) during cotton leaf abscission were selected for further analysis. Our study shows that TDZ-SRGs covered hormone synthesis and signal transduction genes, particularly auxin, cytokinin, and brassinolide, as well as auxin transport genes in leaves, to control cotton leaf abscission. In addition, both auxin and cytokinin contents significantly decrease after TDZ treatment. The role of auxin transport in TDZ-induced leaf abscission was further confirmed by using *pro35S::GhPIN3a::YFP* transgenic plants and exogenous auxin transport inhibitor treatments. Interestingly, the content of brassinosteroids (BR) significantly increased after TDZ treatment, and additional application of BR promoted the TDZ-induced leaf abscission. The findings will not only deepen our understanding of the mechanism of chemical defoliation in cotton, but also provide a theoretical basis for the development of cotton varieties highly suitable to chemical defoliation and guide the formulation of new, efficient defoliants.

## 2. Results

### 2.1. Effect of TDZ and Ethephon (Eth) on Defoliation and Ethylene Production

**Figure 1:**
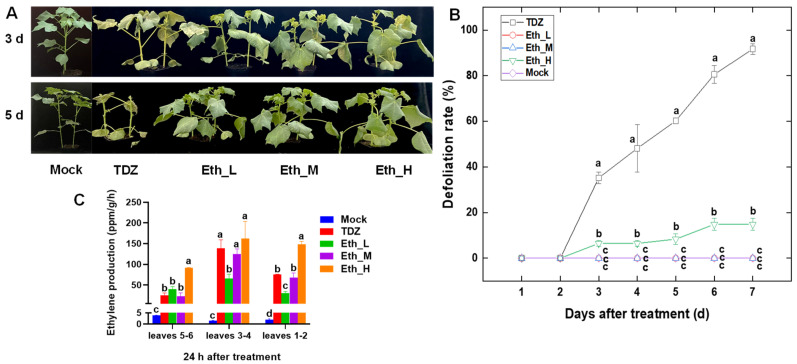
Effect of thidiazuron (TDZ) and ethephon (Eth) on cotton leaf defoliation and ethylene production. (**A**) Representative plants at 3 d and 5 d after treatment with 300 mg/L TDZ, or ethephon with 0.2 (Eth_L), 0.6 (Eth_M), and 1.2 (Eth_H) mg/L. (**B**) Defoliation rate (%) over time after treatment. (**C**) Ethylene production of upper (5-6), middle (3-4), and lower (1-2) leaves at 24 h after treatment. Error bars represent the SD of three biological replicates. Homogeneous subgroups with significant differences (*p* ≤ 0.05) are marked by different lowercase letters.

Leaf abscission and ethylene production in cotton leaves were compared among water control (Mock), Eth, and TDZ treatments (Figure 1). Treatment with TDZ resulted in significant abscission, which began about 3 d after treatment and gradually increased to almost 100% at 7 d after treatment. However, only a few upper leaves shed in the 1.2 mg/L (Eth_H) ethephon treatment, and leaf abscission did not occur in the 0.2 (Eth_L) and 0.6 (Eth_M) mg/L ethephon treatments (Figure 1A,B). In addition, both TDZ and ethephon treatments stimulated the release of ethylene from the leaf blades 24 h after treatment. As the concentration of ethephon increased, the ethylene production also increased, and the production under Eth_M was similar to that under TDZ (Figure 1C). Therefore, Eth_M and TDZ treatments were selected for transcriptome analysis to further explore the mechanism of TDZ-induced leaf abscission. Moreover, we found that the younger upper leaves (5-6) produced less ethylene than the older leaves (3-4 and 1-2) 24 h after treatments (Figure 1C), but they were usually the first ones to shed after TDZ treatment.

### 2.2. Effect of TDZ and Eth on Phytohormones Content in Leaves

**Figure 2:**
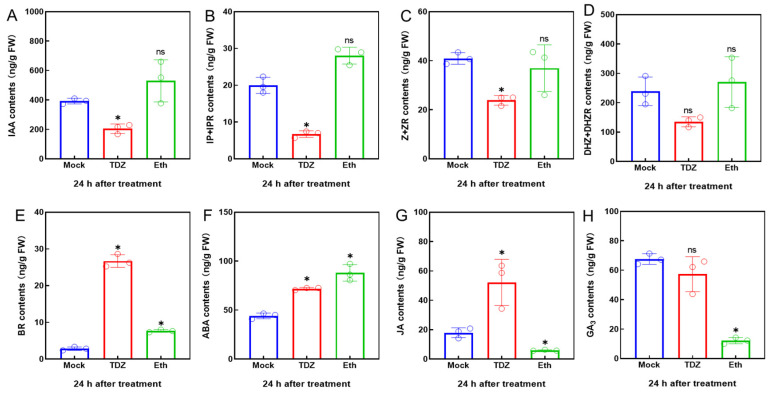
The content of auxin (IAA, (**A**)), isopentenyl adenine and isopentenyl adenosine (IP+IPR, (**B**)), zeatin and zeatin riboside (Z+ZR, (**C**)), dihydrozeatin and dihydrozeatin riboside (DHZ+DHZR, (**D**)), brassinosteroid (BR, (**E**)), abscisic acid (ABA, (**F**)), jasmonic acid (JA, (**G**)), and gibberellin (GA_3_, (**H**)) in the fourth leaf (from bottom) of cotton plants at 24 h after treatment with 300 mg/L thidiazuron (TDZ) or 0.6 mg/L ethephon (Eth). Mock: water. Significant differences between TDZ or Eth and Mock were determined by *t*-test, * indicates *p*-value < 0.05, ns indicates no significant difference.

The levels of auxin (IAA), isopentenyl adenine and isopentenyl adenosine (IP+IPR), zeatin and zeatin riboside (Z+ZR), dihydrozeatin and dihydrozeatin riboside (DHZ+DHZR), brassinosteroid (BR), abscisic acid (ABA), jasmonic acid (JA), and gibberellin (GA_3_) in the fourth leaf from the bottom at 24 h after TDZ treatment are shown in Figure 2. The contents of IAA and three types of cytokinin (IPR, ZR, and DHZR), decreased significantly 24 h after TDZ treatment but were not affected by Eth treatment. On the contrary, the contents of BR and JA increased significantly after TDZ treatment, reaching about nine times and three times higher, respectively, and both were higher than the amounts after the Eth treatment. In addition, the ABA content was significantly higher than Mock after TDZ and Eth treatment, while the GA_3_ content decreased only after Eth treatment.

### 2.3. Dynamic Transcriptome Profiles in Leaves during Chemical Defoliation

To determine the specific transcriptional changes in the fourth leaf from bottom in response to TDZ, RNA-seq (totaling 36 libraries) was performed for cultivar SCRC22 at 3, 6, 12, and 24 h after the TDZ and Eth treatments, and water was used as Mock. For each sample, we acquired about 81 million raw reads and at least 74 million clean reads. The clean reads were then mapped to the *G. hirsutum* reference genome. Only the reads that were uniquely mapped, which averaged 86.3% of all reads (Supplementary Table S1), were utilized to determine the normalized gene expression level, which was expressed as FPKM and used for further analysis of DEGs.

**Figure 3:**
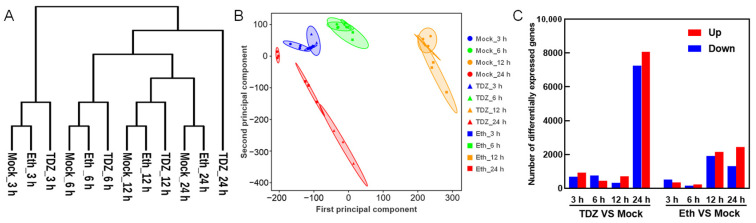
Transcriptome features and relationships among all samples. (**A**) Hierarchical clustering diagram showing the time series distribution of the samples. Cotton plants were treated with 300 mg/L thidiazuron (TDZ) or 0.6 mg/L ethephon (Eth). Mock: water. (**B**) Principal component analysis (PCA) of 36 libraries taken at 4 time points. (**C**) The number of up- and downregulated differentially expressed genes in cotton leaf upon TDZ or Eth treatment.

The three biological replicates were compared, and it was apparent that they had strong correlations between their expression values (average R > 0.96) (Supplementary Figure S1). Therefore, we determined the expression level for the sample at each time point using the average FPKM value of the three replicates. Principal component analysis (PCA) and hierarchical clustering were used to evaluate the relationships among the transcriptome samples (Figure 3A,B). The results showed that all samples were well grouped by time point and defoliant treatment, indicating a progressive response of gene expression and significant changes in transcripts after TDZ and Eth treatment. We also found that the samples clustered much more significantly in relation to sample timing (3, 6, 12, 24 h) than to type of treatment (mock, Eth, TDZ), possibly because the circadian rhythm appears to affect the plant transcriptome even more noticeably than the TDZ or ethephon treatment.

A total of 19,176 and 9063 DEGs were obtained from the leaves after TDZ and Eth treatments, respectively. Among the four time points, 24 h after treatment was the point with the largest number of DEGs. Compared to Mock, there were more up- and downregulated DEGs in TDZ treatment than in Eth treatment at 3, 6, and 24 h following treatment. By contrast, the number of DEGs in the TDZ treatment was lower than that of the Eth treatment at 12 h (Figure 3C). For upregulated DEGs, there were 862, 426, 463, and 5896, specifically in response to the TDZ at 3, 6, 12, and 24 h, while the downregulated DEGs were 543, 732, 210, and 6217, respectively. After eliminating duplicated DEGs at different time points, a total of 13,764 genes were identified as specific TDZ responsive genes (TDZ-SRGs, Supplementary Figure S2).

### 2.4. Temporal Expression Pattern of TDZ-SRGs in Leaves

**Figure 4:**
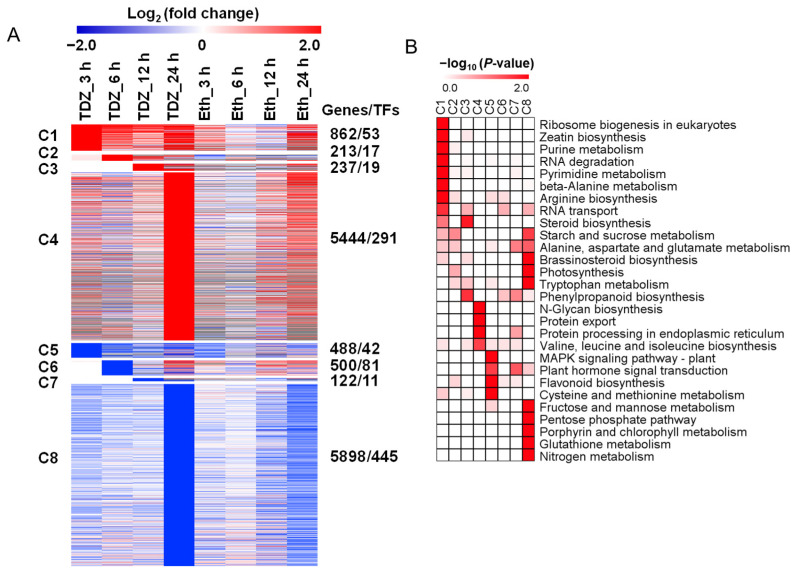
Expression pattern of genes specifically in response to thidiazuron (300 mg/L TDZ) compared with ethephon (0.6 mg/L) (TDZ-SRGs) and functional enrichment analysis over the time course. (**A**) Heat map of the 13,764 TDZ-SRGs over time using log_2_ (fold-change) values of genes. The heat map displays expression patterns of the eight clusters, which are grouped by the start time of each gene’s response to TDZ, with red indicating upregulated expression (C1–C4) and blue indicating downregulated expression (C5–C8). (**B**) KEGG functional categories enriched in C1–C8 gene clusters. Significant categories (*p* < 0.05) were displayed.

All 13,764 TDZ-SRGs were grouped into eight clusters based on the onset of TDZ response (Figure 4A), where upregulated genes (6756 genes, including 380 TFs) were separated into C1–C4 clusters and downregulated genes (7008 genes, including 578 TFs) were separated into C5–C8 clusters. KEGG pathway analysis was then performed to assign genes to biological pathways for each cluster (Figure 4B).

The TDZ-SRGs that responded rapidly after 3 h of TDZ treatment could be divided into two types: upregulated (C1 cluster) and downregulated (C5 cluster) which contained 862 genes (including 53 TFs) and 488 genes (including 42 TFs), respectively (Figure 3A). Genes in the C1 cluster were mainly enriched in “Ribosome biogenesis in eukaryotes”, “Zeatin biosynthesis”, “Purine metabolism”, “RNA degradation”, “Pyrimidine metabolism”, “Beta-alanine metabolism” and “Arginine biosynthesis” pathway. Meanwhile “MAPK signaling pathway—plant”, “Plant hormone signal transduction”, “Flavonoid biosynthesis”, “Cysteine and methionine metabolism” and “Fructose and mannose metabolism” were significantly dominant in the C5 cluster (Figure 4B).

The upregulated genes that started to respond to TDZ at 6 h after treatment were best represented by the C2 cluster, which included 213 expressed genes with 17 TFs. In this cluster, genes were mainly enriched in the “Starch and sucrose metabolism” and “Photosynthesis” pathways. In addition, 500 genes (C6 cluster, including 81 TFs) began to be specifically downregulated at 6 h after TDZ treatment, but no significant pathway was enriched by KEGG analysis. At 12 h after treatment, a total of 359 TDZ-SRGs, including 30 TFs in the C3 and C7 clusters, began to respond to TDZ. KEGG enrichment analysis indicated that genes annotated to “Steroid biosynthesis” and “Phenylpropanoid biosynthesis” were upregulated, while genes annotated to “Plant hormone signal transduction” and “Alanine, aspartate and glutamate metabolism” were downregulated.

As shown in Figure 4A, major TDZ-SRGs began to respond to TDZ at 24 h after treatment, which contained 5444 upregulated genes (including 291 TFs) and 5898 downregulated genes (including 445 TFs) in C4 and C8 clusters, respectively. The KEGG enrichment results indicated that genes related to “N-glycan biosynthesis”, “Protein export”, “Protein processing in endoplasmic reticulum” and “Valine, leucine and isoleucine biosynthesis” were upregulated, while those related to “Starch and sucrose metabolism”, “Alanine, aspartate and glutamate metabolism”, “Brassinosteroid biosynthesis”, “Photosynthesis”, “Tryptophan metabolism”, “Plant hormone signal transduction”, “Fructose and mannose metabolism”, “Pentose phosphate pathway”, “Porphyrin and chlorophyll metabolism”, “Glutathione metabolism”, and “Nitrogen metabolism” were downregulated.

### 2.5. TDZ-SRGs in Cotton Leaves Involved in Phytohormone Signal Transduction and Hormone Synthesis Pathways

**Figure 5:**
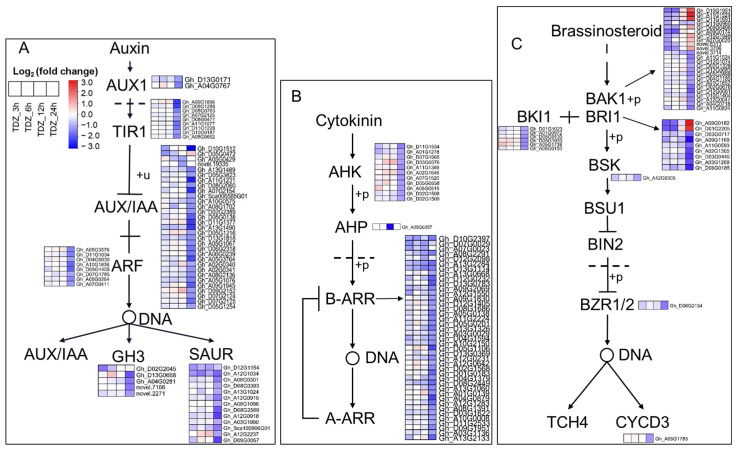
Genes with specific response to 300 mg/L thidiazuron (TDZ) compared with 0.6 mg/L ethephon (TDZ-SRGs) involved in plant hormone signal transduction. (**A**) Auxin. (**B**) Cytokinin. (**C**) Brassinosteroid.

Plant hormones play an important role in regulating a variety of developmental processes, including organ abscission. In total, we found 258 TDZ-SRGs to be involved in “Plant hormone signal transduction” pathway in cotton leaves, of which 32 were in cluster C5, 13 in C7, and 213 in C8. Based on the number of TDZ-SRGs, the most important hormones were auxin (IAA) and cytokinin (CTK), followed by brassinosteroid (BR), gibberellin (GA), abscisic acid (ABA), ethylene, jasmonic acid (JA), and salicylic acid (Figure 5 and Supplementary Figure S3). In total, 68 auxin signaling-related genes were found to be specifically regulated by TDZ. Almost all of them were significantly downregulated 24 h after treatment, including two genes encoding auxin influx carrier (*AUX1*), nine genes encoding auxin receptor TIR1, 29 genes of *AUX/IAA*, eight auxin response factors (*ARFs*), four GH3 protein and 13 SAUR family protein. Fifty-three cytokinin signaling related genes showed decreased expression under TDZ treatment, including 11 cytokinin receptor genes (*AHK*), one histidine-containing phosphotransfer protein gene (*AHP*), and 41 type-B cytokinin response regulators (*B-ARRs*). In total, 10 of the 42 brassinosteroid signaling related genes were upregulated 24 h after TDZ treatment, including 8 *BAK*1 and 2 *BRI1* genes; the others were repressed by TDZ, including *BAK1*, *BRI1*, *BKI1*, *BSK*, *BZR1/2*, and *CYCD3*.

**Figure 6:**
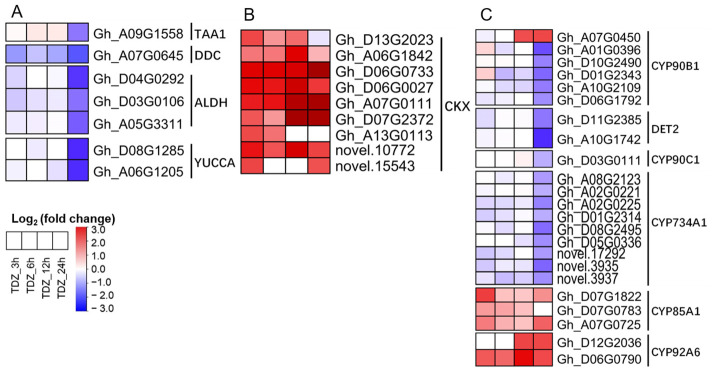
Genes with specific response to 300 mg/L thidiazuron (TDZ) compared with 0.6 mg/L ethephon (TDZ-SRGs) involved in auxin (**A**), cytokinin (**B**) and brassinosteroid (**C**) synthesis and/or metabolism pathways.

In addition to “Plant hormone signal transduction”, “Tryptophan metabolism”, “Zeatin biosynthesis”, and “Brassinosteroid biosynthesis” pathways related to hormone synthesis and/or metabolism were also significantly enriched (Figure 6). In the tryptophan (Trp)-dependent auxin biosynthesis pathway, a total of seven genes encoding four key enzymes were downregulated, including Trp aminotransferase (TAA1), dopa decarboxylase (DDC), acetaldehyde dehydrogenase (ALDH), and flavin monooxygenases (YUCCA). In the KEGG database, cytokinin oxidase/dehydrogenase (CKX) genes that could reduce the endogenous cytokinin content in plants are mistakenly classified under the “Zeatin biosynthesis” pathway. Our KEGG enrichment analysis results indicated that nine CKX genes were inaccurately classified within the “Zeatin biosynthesis” pathway and showed significantly upregulated by TDZ. For the “Brassinosteroid biosynthesis” pathway, a total of 23 genes were identified, most of which were members of cytochrome P450 enzymes (CYPs). Among them, one gene encoding CYP90B1, two genes encoding CYP92A6, and three genes encoding CYP85A1 were upregulated, while the remaining five genes encoding CYP90B1, two genes encoding 5a-reductase (DET2), nine genes encoding CYP734A1, and one gene encoding CYP90C1 were downregulated.

### 2.6. Effect of Exogenous BR Application on TDZ-Induced Leaf Abscission

**Figure 7:**
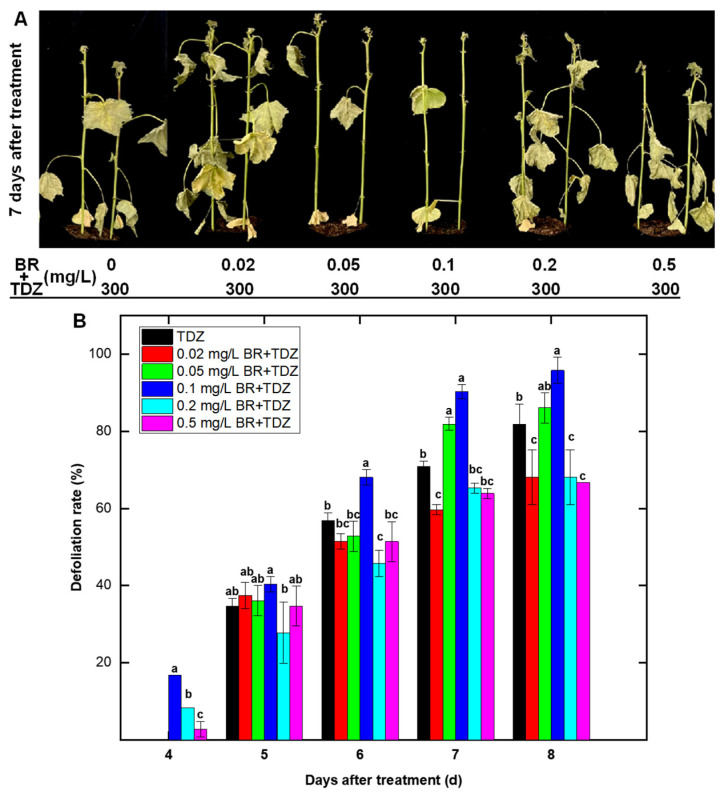
Effect of brassinosteroid (BR) pretreatment (2 h in advance) on thidiazuron (TDZ)-induced cotton leaf abscission. (**A**) Representative plants at 7 d after treatments. (**B**) Defoliation rate (%) over time after treatments. Homogeneous subgroups with significant difference (*p*-value < 0.05) are marked by different lowercase letters.

To clarify the effect of BR on TDZ-induced leaf abscission, cotton plants were pretreated with different concentrations of BR before TDZ application. As shown in Figure 7, 0.05 mg/L and 0.1 mg/L BR effectively promoted cotton leaf drop induced by TDZ. Compared with TDZ treatment alone, the defoliation rate of these two BR pretreatment increased by 4.2% and 13.9%, respectively, at 8 d after treatment.

### 2.7. TDZ-SRGs in Leaves Associated with Auxin Transport

**Figure 8:**
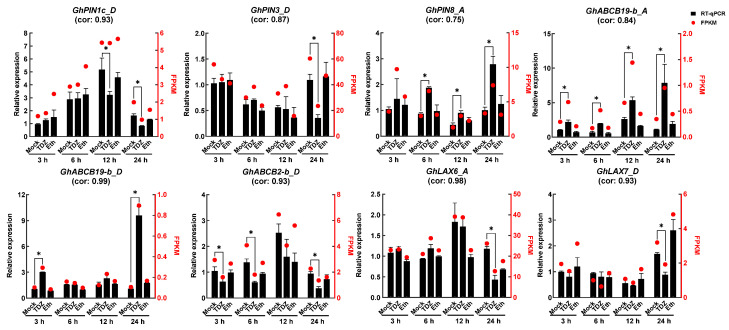
Expression profiles of genes with specific response to 300 mg/L thidiazuron (TDZ) compared with 0.6 mg/L ethephon (TDZ-SRGs) related to auxin transport. Pearson’s correlation coefficient was used to estimate the agreement (cor) between the RT-qPCR expression levels and RNA-seq data. Red dots indicate FPKM value from RNA-seq. Significant differences between TDZ or Eth and Mock were determined by *t*-test, * indicates *p*-value < 0.05.

Auxin transport regulated the distribution of auxin between the leaf and the stem, which is critical for TDZ-induced leaf abscission [27]. In all TDZ-SRGs, eight genes were found related to auxin transport, including *GhPIN1-c_D*, *GhPIN3_D*, *GhPIN8_A*, *GhABCB19-b_A*, *GhABCB19-b_D*, *GhABCB2-b_D*, *GhLAX6_A*, and *GhLAX7_D* (Figure 8). Most of the genes showed specific responses at 24 h after TDZ treatment, with five genes significantly downregulated and the remaining genes (*GhPIN8_A*, *GhABCB19-b_A* and *GhABCB19-b_D*) upregulated. In addition, *GhABCB19-b_A* and *GhABCB2-b_D* were 2 genes that responded quickly to TDZ, and the response continued until 24 h after treatment, showing continuous upregulation and downregulation, respectively, starting from 3 h after treatment. The expression levels of these genes were confirmed using RT-qPCR, which provided similar results to the RNA-seq data, indicating that our data were reliable.

### 2.8. Effect of Auxin Transport on Chemical Defoliation Induced by TDZ

**Figure 9:**
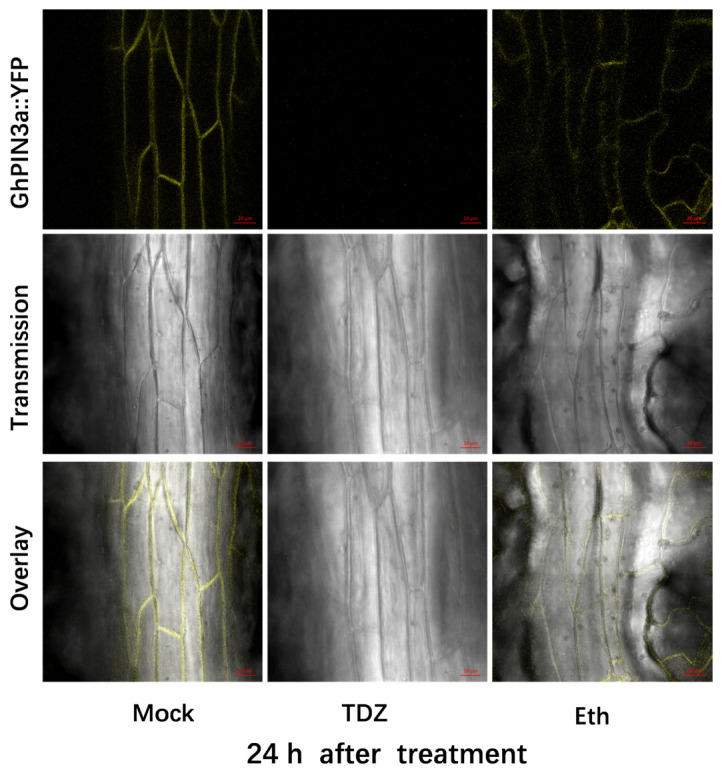
Effect of thidiazuron (TDZ, 300 mg/L) and ethephon (Eth, 0.6 mg/L) on *GhPIN3a::YFP* expression and protein localization in vein epidermal cells of the fourth leaf from bottom of cotton plants at six-leaf stage.

To further investigate the specific responses of auxin transport to TDZ, the *pro35S::GhPIN3a::YFP* transgenic cotton plants were treated with TDZ or Eth. Fluorescence images showed that the *GhPIN3a::YFP* signal appeared at the plasma membrane of vein epidermal cells, and the YFP signal almost completely disappeared in the vein epidermal cells 24 h after TDZ treatment, while a relatively weak signal could still be seen in Eth treatment (Figure 9).

**Figure 10:**
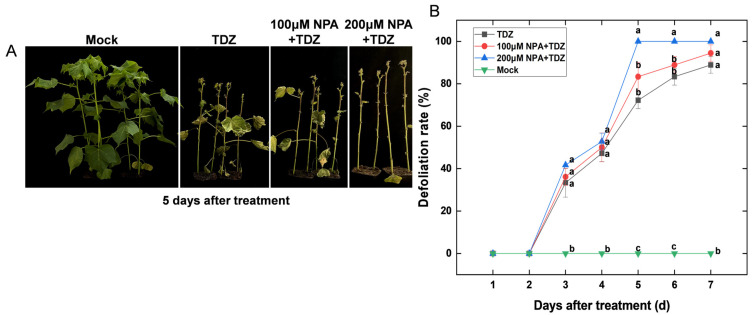
The importance of auxin transport in thidiazuron (TDZ)-induced leaf abscission. (**A**) Representative plants taken at 5 d after treatment with 300 mg/L TDZ, or TDZ plus 100 and 200 μM 1-naphthylphthalamic acid (NPA), respectively. (**B**) Defoliation rate (%) of cotton leaves over time. Error bars represent the SD of three biological replicates. Homogeneous subgroups with significant difference (*p*-value < 0.05) are marked by different lowercase letters.

In addition, we treated cotton plants with 300 mg/L TDZ, or TDZ plus 100 and 200 μM 1-naphthylphthalamic acid (NPA, an inhibitor of auxin transport) (Figure 10). The leaves began to fall off 3 days after treatment, but there was no significant difference among treatments until 4 days after treatment. However, the defoliation rate of TDZ plus 200 μM NPA reached 100 percent 5 days after treatment, which was significantly higher than the rate of other treatments. Except for the Mock treatment, the defoliation rate of TDZ treatment alone was the lowest at all time points after treatment. These results suggested that inhibiting auxin transport could effectively promote TDZ-induced cotton leaf abscission.

### 2.9. TDZ-SRGs Encoding for Transcription Factors (TFs) in Cotton Leaves

**Figure 11:**
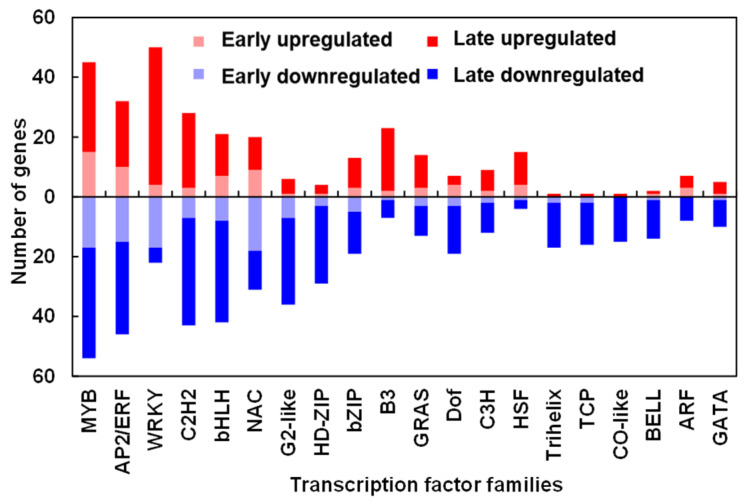
Summary of the top transcription factor families in the genes specifically responsive to 300 mg/L thidiazuron (TDZ) compared with 0.6 mg/L ethephon (TDZ-SRGs). Early: 3, 6, and 12 h after treatment; Late: 24 h after treatment. Red indicates upregulated expression and blue indicates downregulated expression.

Transcription factors are crucial regulatory elements that influence the expression of functional genes and participate in various plant growth and development processes. The 959 transcription factors (TFs) that specifically responded to TDZ treatment originated from 56 families, and the top 20 families are shown in Figure 11. In general, TFs mostly responded at a later stage (24 h) upon TDZ treatment, and more TFs were downregulated than upregulated. Among them, the most upregulated TF families in the early stage (3, 6, and 12 h) were *MYB*, *AP2/ERF*, *NAC*, and *bHLH,* with 15, 10, 9, and 7 genes, respectively. *WRKY*, *MYB*, *C2H2*, and *AP2/ERF* were the most upregulated TF families at the late stage, with 46, 30, 25, and 22 members, respectively. In addition, the largest number of TFs downregulated in the early stage were from the *NAC*, *WRKY*, *MYB*, and *AP2/ERF* families, with 18, 17, 17, and 15, respectively. Moreover, the largest TF family downregulated in the late stage was *MYB* (37 genes), *C2H2* (36 genes), *bHLH* (34 genes), and *AP2/ERF* (31 genes). These findings suggested that genes from these TF families might be involved in initiating the transcriptional cascade during TDZ-induced cotton leaf abscission.

### 2.10. Co-Expression Network Analysis Associated with TDZ Treatment

**Figure 12:**
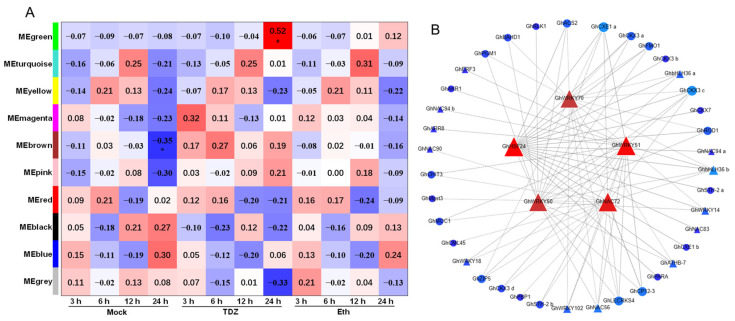
Gene co-expression network analysis by WGCNA. (**A**) Module–sample phenotype association, * indicates *p*-value < 0.05. (**B**) Network of Hub transcription factors in green module. Triangles represent transcription factors, circles represent functional genes, red represents hub transcription factors within modules, and blue represents of hub transcription factors target genes.

To investigate the regulatory network and determine key genes involved in TDZ-induced cotton leaf abscission, weighted gene co-expression network analysis (WGCNA) was performed. The 10 different co-expression modules corresponding to clusters of associated transcripts were identified (Figure 12A). Among them, the “green” module comprising 1974 genes (including 168 TFs) was most highly correlated with the sample phenotype at 24 h after TDZ treatment. To further explore the transcriptional mechanisms of leaf abscission, we used Cytoscape software version 3.9.1 to visualize the network of the five TFs with top WGCNA edge weight in “green” modules (Figure 12B). In the center of the network diagram, five hub TFs were presented, including *GhNAC72*, *GhWRKY51, GhWRKY70*, *GhWRKY50*, and *GhHSF24*. The outer layer of network consisted of 38 genes (including 13 TFs). Interestingly, several cytokinin related genes were identified in the outer layer of the network, including five *GhCKX* genes and one *GhARR* gene, indicating that cytokinin played an important role in TDZ-induced cotton leaf abscission. Additionally, three out of the five *GhCKX* genes in the outer layer, *GhCKX3a (Gh_D07G2372)*, *GhCKX3b (Gh_D06G0733)*, and *GhCKX3c (Gh_A07G0111)*, were identified as TDZ-SRGs and showed significantly upregulated expression levels within 3–24 h after TDZ treatment (Figure 6).

## 3. Discussion

Chemical defoliation is a crucial agronomic practice for the mechanical harvesting of cotton, as it significantly influences both harvesting efficiency and fiber quality [31]. Thidiazuron is the primary active component of hormonal defoliants utilized in cotton production [32]. It has been reported that disruption of hormone homeostasis in cotton leaves is the main mechanism of TDZ-induced leaf abscission [29,30], especially in stimulating ethylene production and affecting auxin transport in leaves [26,27]. Our study confirmed these previous results, and also generated new discoveries.

### 3.1. The Release of Ethylene from Leaves Was Not a Sufficient Condition for Chemical Defoliation

Our results indicated that the treatment with 0.6 mg/L ethephon did not result in leaf abscission in cotton (Figure 1), despite the ethylene release levels being similar to those treated with 300 mg/L TDZ. Morgan et al. [33] and Xu et al. [28] reported similar results; in their study, ethephon treatment alone did not result in a higher defoliation rate in well-watered cotton plants. These results suggest that ethylene is more likely to play a synergistic rather than dominant role in TDZ-induced defoliation. In addition, we found that the young upper leaves were the first to shed in response to TDZ treatment, but that it was not related to ethylene release, indicating that factors other than ethylene production are involved in TDZ-induced leaf abscission.

### 3.2. The Role of Auxin and Cytokinins in TDZ-Induced Cotton Leaf Abscission

Auxin is a crucial phytohormone that regulates organ abscission in plants [5,11]. The major components of auxin signaling are three groups of auxin-response genes *Aux/IAA, SAUR*, and *GH3*, as well as *ARFs* that regulated these auxin-response genes. In roses, downregulation of *RhIAA16* by virus-induced gene silencing promoted petal abscission [34]. In addition, it was previously reported that several *IAA* genes were down regulated during the abscission of tomato flowers [7]. Mutation and expression analysis indicated that *ARF* genes were able to regulate plant organ abscission [35]. The *GH3* genes have also been reported to be involved in flower shedding, resulting in the conversion of the active free indole-3-acetic acid into an inactive conjugated form [36]. The expression of the *GH3* gene was downregulated in the AZ after the removal of tomato leaves, and rapidly responds to exogenous IAA treatment [37]. Our data consistently indicated that several auxin-response genes (including five *GH3* genes) in cotton leaf were downregulated 24 h after TDZ treatment (Figure 5A). We also identified seven auxin synthesis genes that were significantly downregulated in cotton leaves upon TDZ treatment (Figure 6A). These genes may be important targets by which TDZ decreased cotton leaf IAA content and promoted leaf abscission; further experiments are needed to verify this.

The abscission of plant organs was closely linked to the concentration gradient of auxin at the proximal and distal parts of the AZ [12,13]. Auxin transport carriers controlled the auxin concentration gradients and mediated the shedding of plant organs. Previous research has indicated a significant reduction in the ability of cotton petioles to transport auxin polarly in plants treated with TDZ [27]. Here we showed that eight auxin transporters-related genes specifically responded to TDZ compared with Eth. However, their expression patterns were not consistent, which may be due to their different functions, such as regulating auxin influx, efflux, or intracellular homeostasis. These auxin transporter genes might play an important role in TDZ-induced leaf abscission and may also allow us to manipulate leaf abscission by specifically controlling these auxin transport genes. In addition, we have demonstrated that *GhPIN3a* was involved in TDZ-induced leaf abscission. Firstly, the *pro35S::GhPIN3a::YFP* transgenic plants showed a lower defoliation rate than wild type (41.7% vs. 94.4%) at 5 d after TDZ application (Supplementary Figure S4). Secondly, the YPF fluorescence almost disappeared in the epidermal cells of vein treated with TDZ, which still could be observed after Eth treatment (Figure 9).

Cytokinins are plant hormones that have a variety of functions, including promoting cell division, delaying senescence, and inhibiting abscission. Research has shown that, as plants begin to senesce, their levels of cytokinins decrease, which can trigger the process of abscission. However, by applying exogenous cytokinins to senescing tissue, it is possible to slow down or even reverse the process of senescence in some plant species [1]. The cytokinin oxidase/dehydrogenase (CKX) participates in the oxidation and degradation of cytokinins, regulating the cytokinin homeostasis. We found that nine CKX genes were sharply upregulated after the TDZ treatment (Figure 6B). Through WGCNA, we also identified five hub TFs (*GhNAC72*, *GhWRKY51*, *GhWRKY70*, *GhWRKY50*, and *GhHSF24*) showing co-expression with *CKX* genes (Figure 12B). These TFs may be important regulatory components in TDZ-induced leaf abscission via regulation of cytokinin contents. In agreement with this, TDZ specifically induced a decrease in cytokinin content in cotton leaves (Figure 2B–D), which was consistent with the results of Xu et al. [28] and Li et al. [29]. In addition, the expression of cytokinin signal transduction genes, including the receptor and a large number of *B-ARR*, was inhibited by TDZ. Therefore, we suggest that reduced cytokinin levels may contribute to TDZ-induced leaf abscission and that these B-ARRs may be important regulators of leaf abscission.

### 3.3. A New Discovery: Brassinosteroid Was Also Involved in TDZ-Induced Chemical Defoliation of Cotton

In this study, the content of BR in cotton leaves increased significantly after TDZ treatment and was significantly higher than that of Eth treatment (Figure 2), suggesting that BR may also be a key component in promoting TDZ-induced cotton leaf abscission. We also found that a large number of BR-related genes specifically responded to TDZ, including genes related to BR synthesis and signal transduction. Exogenous spraying also confirmed that an appropriate concentration of BR could increase the defoliation rate of TDZ treatment (Figure 7). However, the mechanism by which BR affected this process was not clear. It is more likely that BR has crosstalk with other hormones, such as affecting the auxin transport, as reported in other studies [38]. It has been speculated that BR could promote the senescence of Arabidopsis leaves [39]. We suggest that the appropriate concentration of exogenous brassinosteroids applied to cotton leaves may also accelerate senescence, thereby facilitating TDZ-induced abscission.

### 3.4. Transcription Factors (TFs) in Cotton Leaves Involved in TDZ-Induced Abscission

The TFs play an important role in plant development since they function as primary switches in multiple transcriptional regulatory networks, and their expression dynamics may influence many biological processes both temporally and spatially [40]. According to previous studies, several TFs have been identified in organ abscission processes [4]. Moreover, they were mostly expressed in the AZ and regulated the physiological processes of AZ differentiation or cell wall degradation [41]. Here, we found that a total of 959 TFs were affected by TDZ treatment in cotton leaf blade (Figure 4A), and the top families have been shown in Figure 11. Through gene co-expression network analysis, we found five hub TFs (*GhNAC72*, *GhWRKY51*, *GhWRKY70*, *GhWRKY50*, and *GhHSF24*) involved in TDZ-induced cotton leaf abscission (Figure 12B). *ANAC072*, a homologous gene of *GhNAC72*, has been reported to promote chlorophyll degradation and participate in leaf senescence in Arabidopsis [42]. Moreover, *GhNAC72* has also been found to be highly expressed in yellow leaves in cotton, which may be related to leaf senescence [43]. *GhWRKY51* has been reported to directly activate the expression of the key synthesis gene of salicylic acid (SA) *SID2*, thereby promoting SA accumulation and mediating the balance of growth and immune response in cotton plants [44]. The *WRKY70* expression modulated by SA was positively correlated to the activation of Pathogenesis-Related genes [45]. Therefore, we suggested that the five hub TFs identified by the co-expression network may be capable of modulating the expression of abscission related genes within cotton leaves, thus contributing to the abscission process.

### 3.5. Cotton Leaves Undergo Significant Metabolic Changes within 24 h of TDZ Treatment

In addition to the above genes related to phytohormones and TFs, there was a large number of differentially expressed genes that specifically responded to TDZ compared with Eth (Supplementary Figure S2), which provides other clues for exploring the mechanisms of chemical defoliation. The KEGG enrichment analysis indicated that a considerable number of genes associated with metabolism were impacted by TDZ treatment. Specifically, most metabolic pathways were significantly suppressed 24 h after TDZ treatment, with only a few being activated in the early stages (before 3 h) of treatment, including “Purine metabolism,” “Pyrimidine metabolism,” and “Beta-alanine metabolism,”. This suggested that TDZ treatment may reduce photosynthesis and carbohydrate production capacity in cotton leaves to promote their abscission [46], while quickly activating emergency response mechanisms in order to adapt to stress.

## 4. Materials and Methods

### 4.1. Plant Materials and Growth Conditions

The cotton (*Gossypium hirsutum*) cultivar SCRC22, developed by the Cotton Research Center, Shandong Academy of Agricultural Sciences in China, was used in this study. A transgenic line of *pro35S::GhPIN3a::YFP* was generated as described in Zeng et al. [47]. The seeds were grown in plastic pots (10 cm in diameter and 8.5 cm in depth) containing commercial garden soil. The growth chamber was maintained at 12 h light/12 h dark, 28 °C day/22 °C night, 70–80% relative humidity, and 400 μmol m^−2^ s^−1^ photosynthetic photon flux density.

### 4.2. Defoliation Treatment

All experiments in this study were conducted at six-leaf stage. For comparison of leaf abscission rate and ethylene production, thidiazuron (TDZ) at 300 mg/L, ethephon at 0.2 (Eth_L), 0.6 (Eth_M), and 1.2 (Eth_H) mg/L were sprayed uniformly to the whole plants. The control (Mock) was water application. In other experiments, the concentration of ethephon (Eth) used was 0.6 mg/L. For the brassinosteroid pretreatment experiment, 0.02, 0.05, 0.1, 0.2, and 0.5 mg/L 2,4-epibrassinolide were sprayed uniformly to the whole plants in advance; after 2 h, the surface of leaves became dry, and TDZ was applied at 300 mg/L. For auxin transport inhibitor treatment, 100 and 200 μM 1-naphthylphthalamic acid (NPA) were also sprayed uniformly to the whole plants 2 h before 300 mg/L TDZ treatment. For RNA sequencing (RNA-seq), the fourth leaves from bottom under TDZ, Eth, and Mock treatments were carefully sampled at 3 h, 6 h, 12 h, and 24 h after the treatments. The samples were immediately frozen in liquid nitrogen and stored at −80 °C for further analysis. Three biological replicates were used in each experiment.

### 4.3. Measurement of Ethylene Production and Phytohormone Content

Leaf samples excised from cotton plants 24 h after the Mock, TDZ, Eth_L, Eth_M, and Eth_H treatments were placed in 10 mL airtight vials with a rubber cap. After about 4 h, 1 mL of gas was taken from the vial and injected into the gas chromatograph (GC-2010, Shimadzu, Kyoto, Japan) to measure the content of ethylene, as described in Xue et al. [48]. The content of other plant hormones in the leaves was measured using the ELISA method, as reported in Li et al. [29]. Ethylene production was measured at different positions of the main stem leaves from bottom to top (represented by leaves 1-2, leaves 3-4, and leaves 5-6). Other plant hormones were measured only in leaf 4.

### 4.4. RNA Extraction, cDNA Library Preparation and Sequencing

Total RNA was extracted from the fourth leaf from bottom at six-leaf stage using the EASYspin Plus Complex Plant RNA Kit (Aidlab Biotech, Beijing, China). RNA quality was assessed using the Agilent 2100 Bioanalyzer (Agilent Technologies, Santa Clara, CA, USA) prior to library construction. A total amount of 1 μg RNA per sample was used as input material for the RNA sample preparations. Sequencing libraries were generated using NEBNext^®^ Ultra^TM^ RNA Library Prep Kit for Illumina^®^ (NEB, San Diego, CA, USA) following manufacturer’s recommendations, and index codes were added to attribute sequences to each sample. The cDNA libraries were sequenced on the Illumina sequencing platform by Metware Biotechnology Co., Ltd. (Wuhan, China).

### 4.5. Analysis of Differentially Expression Genes

By eliminating junction sequences and low-quality sequences, clean reads were obtained. Using HISAT2 [49], all of the clean reads were aligned to the cotton genome (*G. hirsutum* TM-1 (AD) 1) [50]. FPKM (Fragments Per Kilobase Millon Mapped Reads) was used as an indicator to measure transcription or gene expression levels in each sample [51]. Differentially expression analysis was performed using the DEGseq2 (https://bioconductor.org/packages/release/bioc/html/DESeq2.html (accessed on 1 August 2022)). Genes with a *p*-value < 0.05 and |log2 (Fold Change)| ≥ 1 were assigned as differentially expressed genes (DEGs), and their distribution among samples was shown by volcano plot (Supplementary Figure S5) and MA plot (Supplementary Figure S6). To determine the DEGs involved in plant hormone signal transduction and the distribution of DEGs into multiple biological pathways, KEGG (Kyoto Encyclopedia of Genes and Genomes) enrichment analysis was performed using the KOBAS software version 3.0 [52].

### 4.6. Reverse Transcription Quantitative Polymerase Chain Reaction (RT-qPCR) Analysis

RT-qPCR was used to verify candidate genes identified by RNA-seq. cDNA synthesis was carried out according to the manufacturer’s instructions using Oligo d (T) primer and M-MLV reverse transcriptase (Takara, Kusatsu, Japan). RT-qPCR was carried out in an Applied Biosystems 7500 Fast Real-Time PCR System (Applied Biosystems, Waltham, CA, USA) using SYBR^®^ Premix Ex Taq™ (Takara, Kusatsu, Japan). The reaction procedure was set as follows: 95 °C for 30 s, 40 cycles of 95 °C for 5 s, 60 °C for 34 s, and 95 °C for 15 s, 60 °C for 60 s, then 95 °C for 30 s, and finally 60 °C for 10 s. A melting curve was performed from 60 °C to 95 °C to check the specificity of the amplified products. Using *GhActin9* as the internal control, the expression level of each gene was calculated and normalized by the 2^−∆∆CT^ method [53]. Supplementary Table S2 lists the primers used in this work.

### 4.7. Gene Co-Expression Network Analysis

The WGCNA package V1.48 [54] in R [55] was used to analyze gene co-expression networks. Gene dendrograms were colored according to the relationships between gene expression levels and were used to form partition modules. The relationship between each module and the sample phenotype was investigated, and the network was visualized with Cytoscape software version 3.9.1 [56].

### 4.8. Microscopic Observations

The *pro35S::GhPIN3a::YFP* transgenic cotton plants were cultivated until the six-leaf stage. They were treated with 300 mg/L TDZ and 0.6 mg/L Eth, and water was used as Mock. After 24 h of treatments, the fourth leaf from the bottom was manually sliced to observe the YFP fluorescence signal on the epidermis of leaf vein with ZEISS LSM880 (Carl Zeiss, Oberkochen, Germany) confocal laser-scanning microscope. The emission and excitation wavelength were 523–600 nm and 514 nm, respectively, and the laser intensity was 5%.

### 4.9. Statistical Analysis

Statistical analysis was conducted using SPSS 22.0 (SPSS Inc., 335 Chicago, IL, USA). After one-way ANOVA, the mean of Mock and treatments were compared by using Duncan’s multiple range test or Student’s *t*-test. The line and bar graphs were created using Origin 2018 software version 2018 (Origin Lab Co., Northampton, MA, USA) and GraphPad Prism 8 version 8.0 (GraphPad Software Inc., San Diego, CA, USA, 2020). Hierarchical clustering and heatmaps were executed using MeV software version 4.9 (Multi Experiment Viewer 4.9, TIGR, Rockville, MD, USA). Principal Component Analysis (PCA) was conducted using the prcomp function in R [55].

## 5. Conclusions

Both thidiazuron (TDZ) and ethephon (Eth) can enhance the ethylene production of cotton leaves, but only TDZ can efficiently induce leaf shedding. Compared with Mock, TDZ decreased the levels of indole-3-acetic acid (IAA) and cytokinins but increased the levels of brassinosteroids (BR) and jasmonic acid (JA) in cotton leaves, whereas Eth had no effect, less effect, or opposite effects, respectively, on this hormones. RNA-seq identified 13,764 differentially expressed genes specifically responding to TDZ as compared with Eth. The KEGG analysis suggest that the synthesis, metabolism, and signal transduction of IAA, cytokinins, and BR were all involved in TDZ-induced abscission, and the combination of BR and TDZ showed a higher defoliation rate than TDZ treatment alone. The expression of eight genes related to auxin transport in the leaf was specifically regulated by TDZ. The auxin transport inhibitor, N-1-naphthylphthalamic acid (NPA) promoted the defoliation effect of TDZ, and the reduced leaf abscission rate in *pro35S::GhPIN3a::YFP* transgenic cotton plants further confirmed the involvement of auxin transport in TDZ-induced leaf abscission. Moreover, the co-expression network analysis (WGCNA) identified five hub transcriptional factors (*GhNAC72, GhWRKY51, GhWRKY70, GhWRKY50,* and *GhHSF24*) associated with TDZ-induced defoliation.

## Data Availability

The data disclosed in this study can be accessed through the article or Supplementary Materials provided here.

## Supplementary Materials

The following supporting information can be downloaded at: https://www.mdpi.com/article/10.3390/ijms24087590/s1.
