# Supplementary material for: Comparative Physiological and Transcriptomic Mechanisms of Defoliation in Cotton in Response to Thidiazuron versus Ethephon
Source: Int J Mol Sci. 2023 Apr 20;24(8):7590. doi: 10.3390/ijms24087590 (PMC10143250; doi:10.3390/ijms24087590)
Supplement: Supplementary file 1 [file ijms-24-07590-s001.zip › ijms-2316480-supplementary/2023.04.04 Supplementary Figures.pdf]

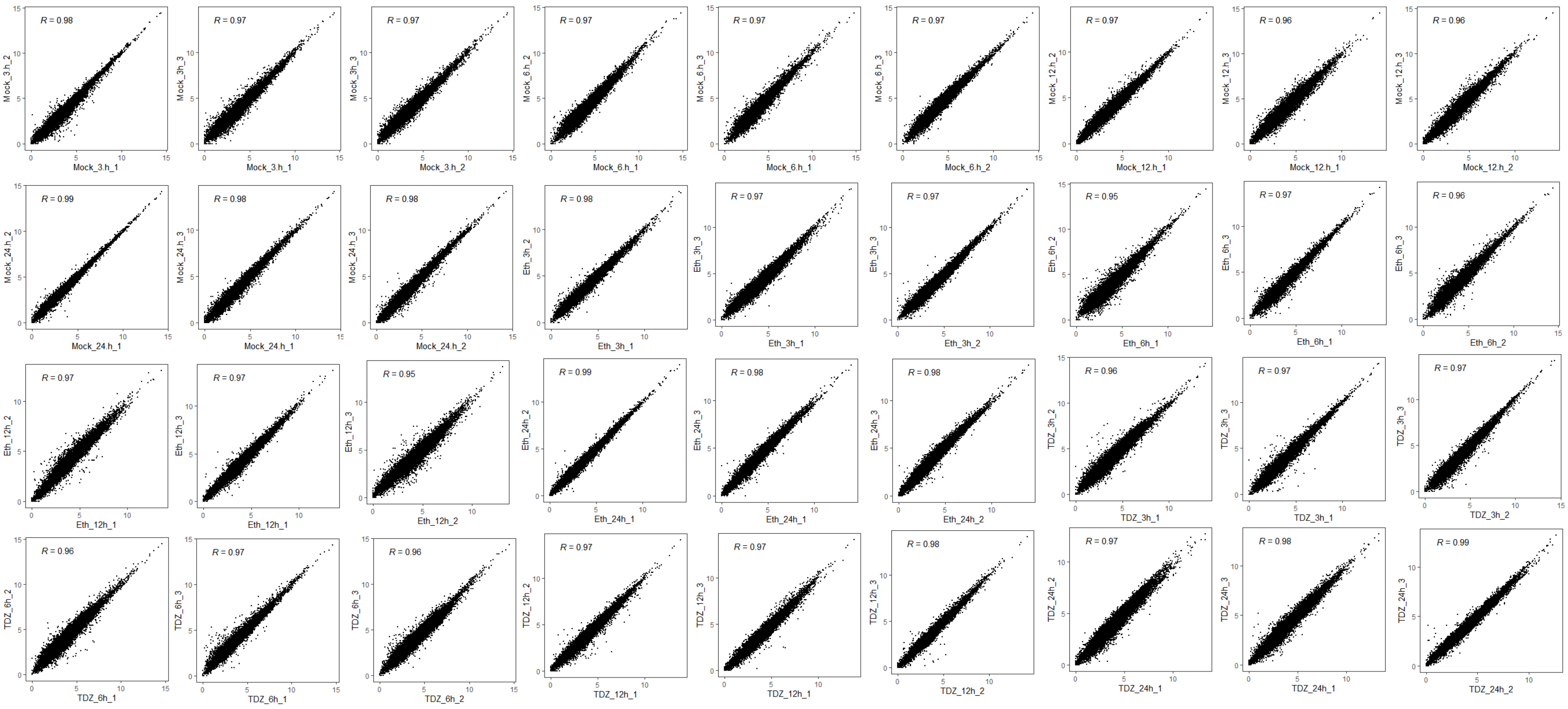

Figure S1. Correlation between biological replicates of samples for RNA-Seq. The correlation coefficient was calculated by using normalized values of log2 (FPKM value + 1). TDZ: 300 mg/L thidiazuron； Eth: 0.6 mg/L ethephon； Mock: water control

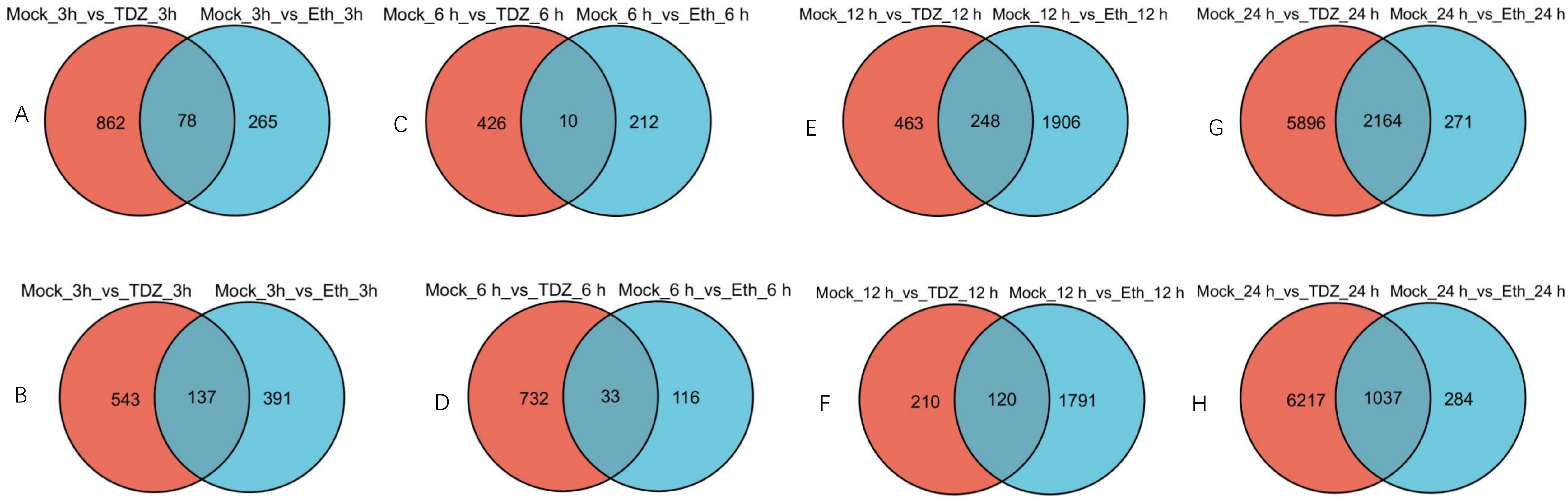

Figure S2. Distribution of differentially expressed genes and identification of TDZ specifically responsive genes. (A), (C), (E), (G): upregulated differentially expressed genes; (B), (D), (F), (H): downregulated differentially expressed genes. TDZ: 300 mg/L thidiazuron； Eth: 0.6 mg/L ethephon； Mock: water control

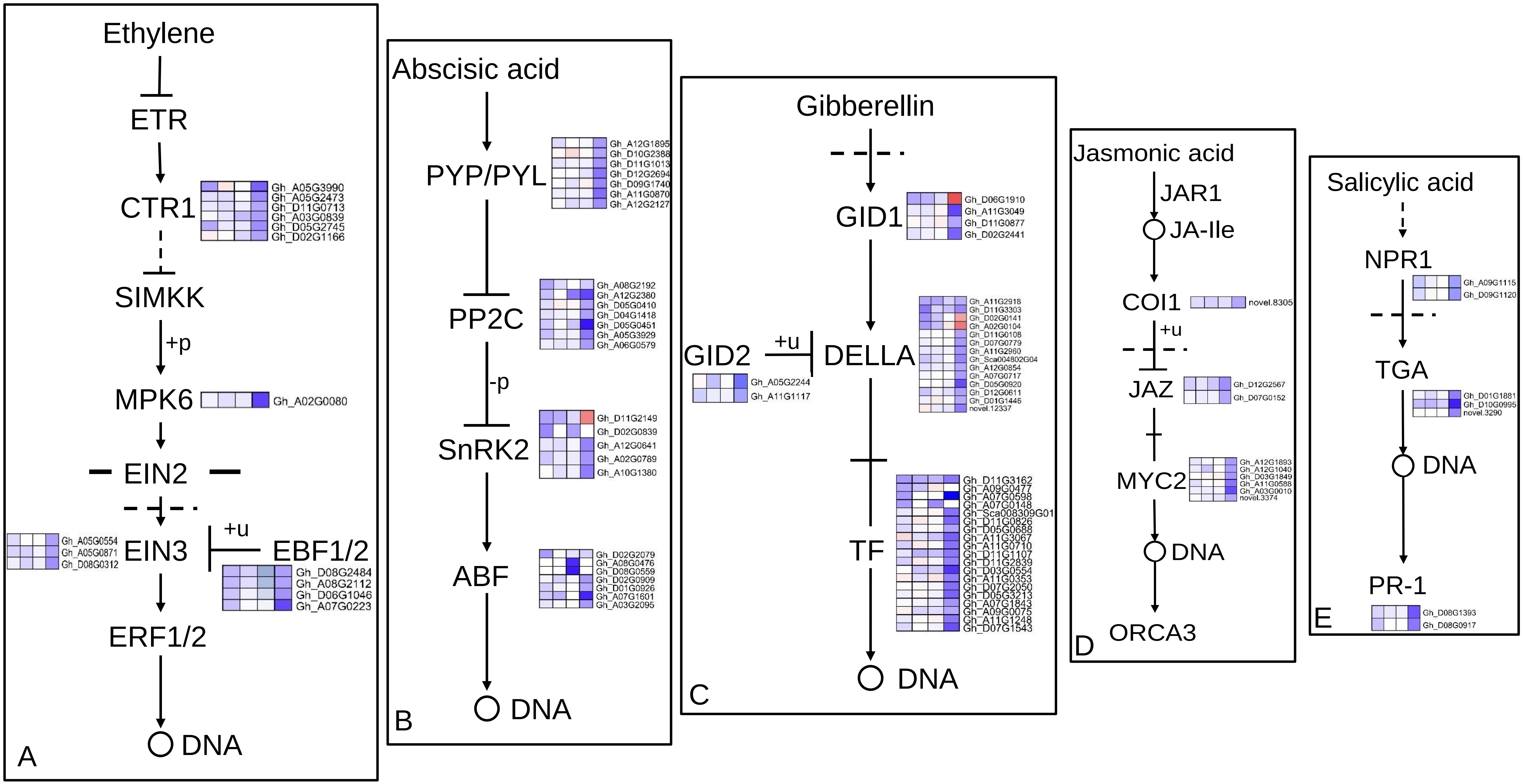

Figure S3. Thidiazuron specifically responsive genes associated with Ethylene (A), abscisic acid (B), gibberellin (C), jasmonic acid (D), and salicylic acid (E) signaling.

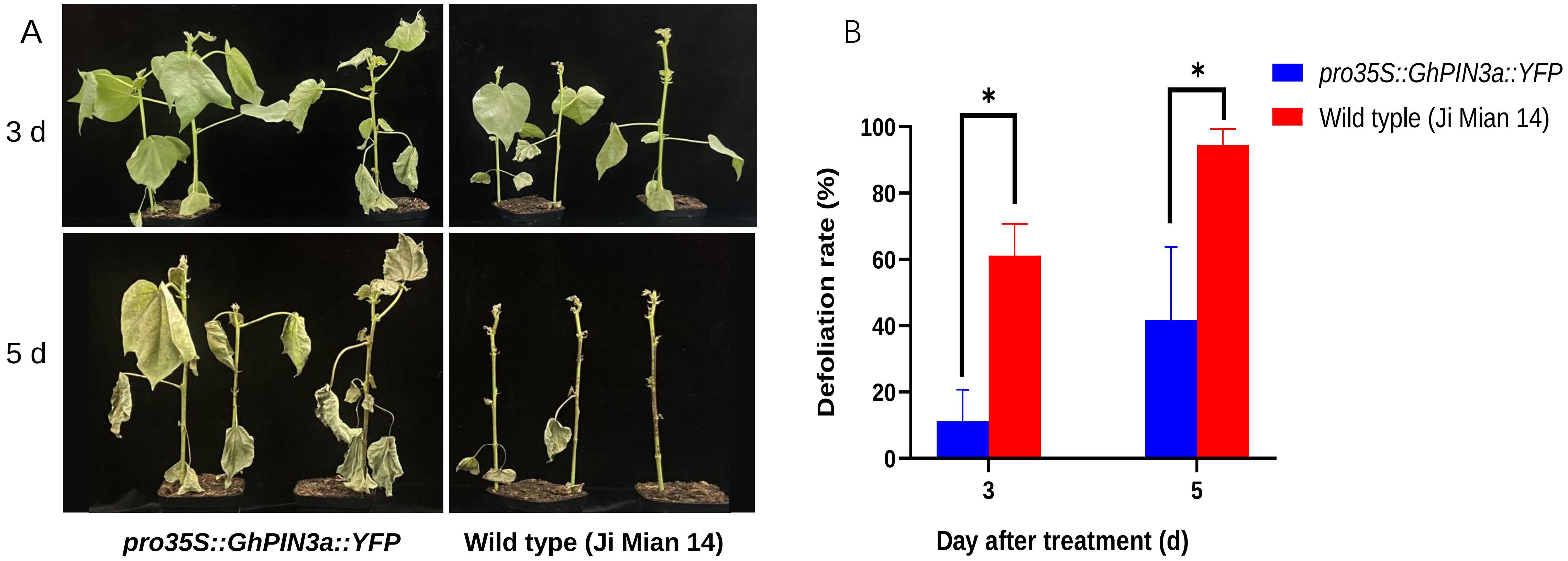

Figure S4. Effect of defoliant treatment on cotton leaf abscission .
(A) Representative images of plants taken at 3 d and 5 d after treatment with 300 mg/L thidiazuron
(B) defoliation rate (%) of cotton leaves at different time points after treatment.

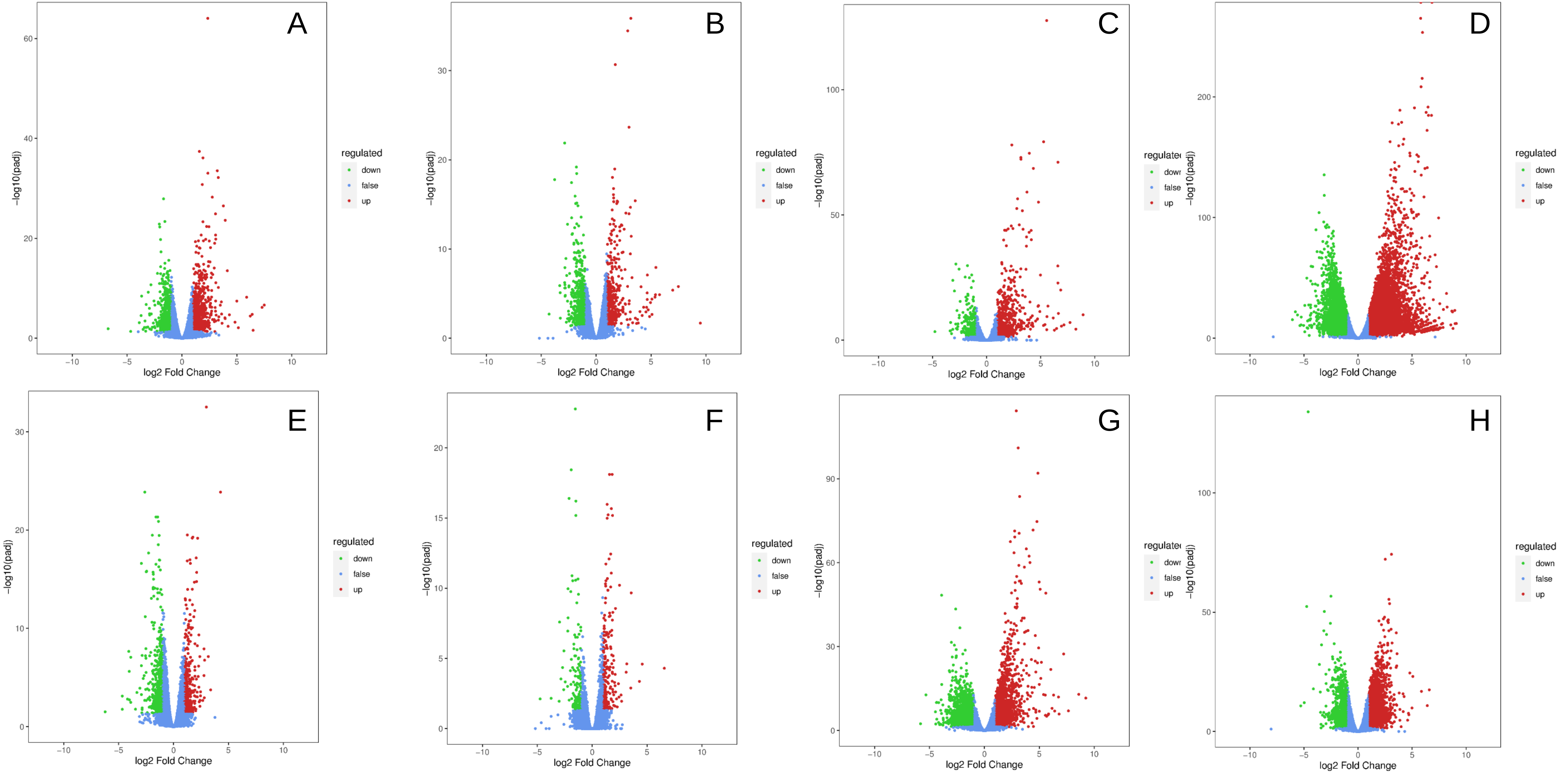

Figure S5. Volcano plot of of differentially expressed genes in different samples.
A-D:TDZ VS Mock; E-H: Eth VS Mock; A&E: 3 HAT; B&F: 6 HAT; C&G: 12 HAT; D&H: 24 HAT.
TDZ: 300 mg/L thidiazuron; Eth: 0.6 mg/L ethephon; Mock: water application; HAT: hours after treatment.

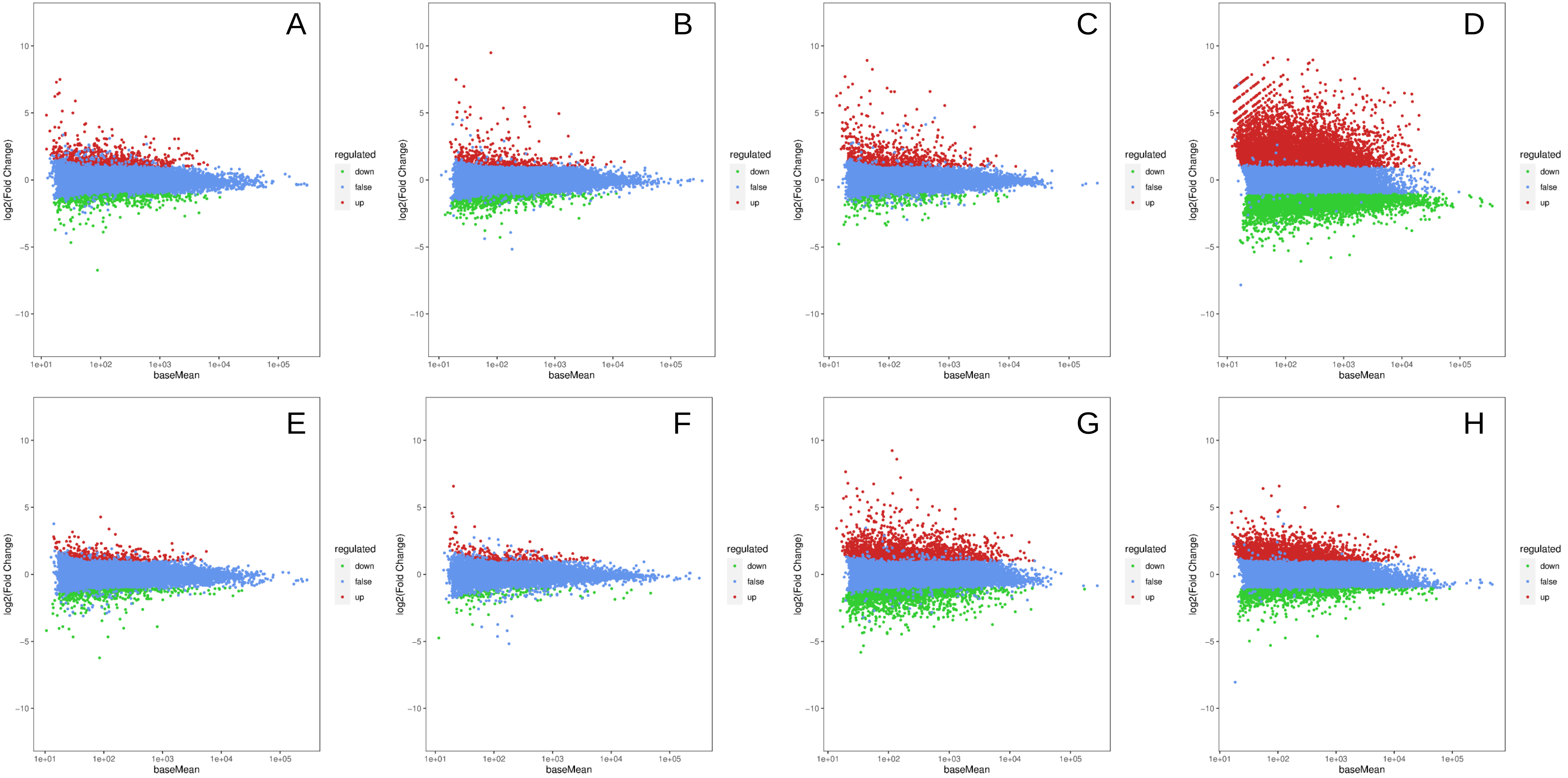

Figure S6. MA plot of of differentially expressed genes in different samples.
A-D:TDZ VS Mock; E-H: Eth VS Mock; A&E: 3 HAT; B&F: 6 HAT; C&G: 12 HAT; D&H: 24 HAT.
TDZ: 300 mg/L thidiazuron; Eth: 0.6 mg/L ethephon; Mock: water application; HAT: hours after treatment.
